# Phylogenetic relationships, origin and historical biogeography of the genus *Sprattus* (Clupeiformes: Clupeidae)

**DOI:** 10.7717/peerj.11737

**Published:** 2021-08-18

**Authors:** Cristian B. Canales-Aguirre, Peter A. Ritchie, Sebastián Hernández, Victoria Herrera-Yañez, Sandra Ferrada Fuentes, Fernanda X. Oyarzún, Cristián E. Hernández, Ricardo Galleguillos, Gloria Arratia

**Affiliations:** 1 Centro i∼mar, Universidad de Los Lagos, Puerto Montt, Chile; 2 Núcleo Milenio INVASAL, Concepción, Chile; 3 School of Biological Sciences, Victoria University of Wellington, Wellington, New Zealand; 4 Biomolecular Laboratory, Center for International Programs, Universidad Veritas, San José, Costa Rica; 5 Sala de Colecciones Biológicas, Facultad de Ciencias del Mar, Universidad Católica del Norte, Coquimbo, Chile; 6 Laboratorio de Genética y Acuicultura, Departamento de Oceanografía, Facultad de Ciencias Naturales y Oceanográficas, Universidad de Concepción, Concepción, Chile; 7 Centro de Investigación en Biodiversidad y Ambientes Sustentables (CIBAS), Universidad Católica de la Santísima Concepción, Concepción, Chile; 8 Instituto Milenio en Socioecología Costera, Santiago, Chile; 9 Laboratorio de Ecología Evolutiva y Filoinformática, Departamento de Zoología, Facultad de Ciencias Naturales y Oceanográficas, Universidad de Concepción, Concepción, Chile; 10 Universidad Católica de Santa María, Arequipa, Perú; 11 Biodiversity Institute and Department of Ecology & Evolutionary Biology, University of Kansas, Lawrence, United States of America

**Keywords:** Antitropical distribution, Sprat, Molecular clock, *Clupea*, BEAST

## Abstract

The genus *Sprattus* comprises five species of marine pelagic fishes distributed worldwide in antitropical, temperate waters. Their distribution suggests an ancient origin during a cold period of the earth’s history. In this study, we evaluated this hypothesis and corroborated the non-monophyly of the genus *Sprattus*, using a phylogenetic approach based on DNA sequences of five mitochondrial genome regions. *Sprattus sprattus* is more closely related to members of the genus *Clupea* than to other *Sprattus* species. We also investigated the historical biogeography of the genus, with the phylogenetic tree showing two well-supported clades corresponding to the species distribution in each hemisphere. Time-calibrated phylogenetic analyses showed that an ancient divergence between Northern and Southern Hemispheres occurred at 55.8 MYBP, followed by a diversification in the Oligocene epoch in the Northern Hemisphere clade (33.8 MYBP) and a more recent diversification in the Southern Hemisphere clade (34.2 MYBP). Historical biogeography analyses indicated that the most recent common ancestor (MRCA) likely inhabited the Atlantic Ocean in the Southern Hemisphere. These results suggest that the ancestral population of the MRCA diverged in two populations, one was dispersed to the Northern Hemisphere and the other across the Southern Hemisphere. Given that the Eocene was the warmest epoch since the Paleogene, the ancestral populations would have crossed the tropics through deeper cooler waters, as proposed by the isothermal submergence hypothesis. The non-monophyly confirmed for the genus *Sprattus* indicates that its systematics should be re-evaluated.

## Introduction

Antitropical distribution patterns—when closely related taxa have geographic distributions to the north and south of the tropics, but not within—are an active line of research in evolutionary biogeography that can benefit greatly from using congeneric species in phylogenetic context. Congeneric species share a common history from their ancestral population, and several studies have shown that the combined analyses of biogeographic history and time-calibrated phylogenies in congeneric species provide a greater insight into the evolutionary processes involved (e.g., Lavoué et al., 2013). There are still important ecological and commercial fish genera with antitropical distribution patterns that remain to be studied, such as the genus *Sprattus*.

**Figure 1:**
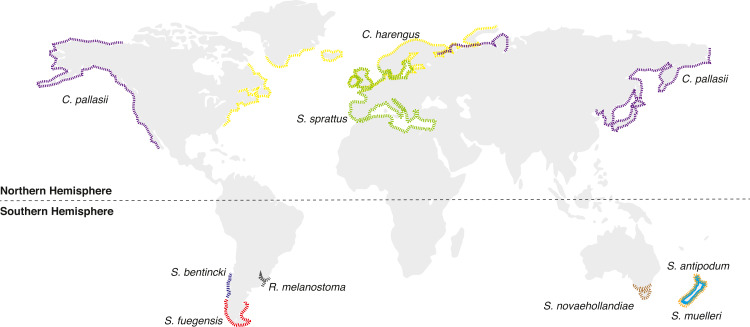
Distributional map of extant *Sprattus* and closely related species used in this study. Red dashed line represents *S prattus fuegensis*; brown is S. *novaehollandiae*; orange is *S*. *muelleri*; light blue is *S*. *antipodum*; and green is *S*. *sprattus*. Yellow solid line represents *Clupea harengus*; purple is *C*. *pallasii*; gray is *Ramnogaster melanostoma*; and blue is *Strangomera bentincki*.

The five extant species currently assigned to the genus *Sprattus* (Fig. 1; Clupeiformes, Clupeidae, Clupeinae) are small marine pelagic fishes that inhabit coastal areas and are well known for their schooling behavior (Whitehead, 1988; Fricke, Eschmeyer & Van der Laan, 2021). They are important components of several food webs and some species are commercially important (Frederiksen et al., 2006). These species occur in cooler waters and have an antitropical distribution (Whitehead, 1988; Fig. 1). *Sprattus sprattus* (Linnaeus, 1758) is the most widely distributed species, and it is the only species in the genus found in the Northern Hemisphere, mainly around the coasts of Europe (Whitehead, 1988; Fricke, Eschmeyer & Van der Laan, 2021). *Sprattus fuegensis* (Jenyns, 1842) is found on the South American coast, mainly in the Patagonian shelf from the Pacific and Atlantic Oceans (Whitehead, 1988; Aranis et al., 2007; Canales-Aguirre et al., 2016; Fricke, Eschmeyer & Van der Laan, 2021). The other three species are found in Oceania: *S. novaehollandiae* (Valenciennes, 1847) in south-eastern Australia, and *S. antipodum* (Hector, 1872) and *S. muelleri* (Klunzinger, 1879) on the coast of New Zealand (Whitehead, Smith & Robertson, 1985; Whitehead, 1988; Fricke, Eschmeyer & Van der Laan, 2021).

Phylogenetic analyses have shown that the genus *Sprattus* is sister to the genus *Clupea* (Lavoué et al., 2007; Li & Ortí, 2007), and it has been suggested that they diversified between 2.66–6.75 MYBP (Jérôme et al., 2003; Cheng & Lu, 2006), which is consistent with the Miocene record of *Clupea*. Moreover, the extant *Clupea* species are thought to have radiated during the Pliocene (3.3–3.5 MYBP; Grant, 1986; Wilson, Teugels & Meyer, 2008), which is when the genus *Sprattus* is thought to have diverged. More recent studies based on large fossil-calibrated phylogenies suggested that the genus *Sprattus* is a paraphyletic group, and *S. sprattus* is more closely related to *Clupea* spp. than to its relatives in the Southern Hemisphere (Lavoué et al., 2013; Bloom & Lovejoy, 2014; Egan et al., 2018).

No study has examined the biogeographic origin of the genus *Sprattus*; though, information of species with similar antitropical distribution pattern have been conducted. For example, studies of extant populations of *Sardinops* species showed a recent diversification event between 0.2–2 MYBP (Grant & Leslie, 1996; Bowen & Grant, 1997; Grant & Bowen, 1998), whereas species included in the genus *Engraulis* diversified between 5–10 MYBP (Grant, Leslie & Bowen, 2005). When considering marine species that have an antitropical distribution, the tropical zone appears to act as a barrier to long-distance dispersal, restricting gene flow between the Northern and Southern Hemispheres (Grant, Lecomte & Bowen, 2010). Experimental studies aiming to evaluate the thermal tolerance of two temperate species of Clupeidae (e.g., *Clupea harengus* and *Sardinops sagax*) evidenced their low tolerance for warm (tropical) waters (Martínez-Porchas, 2009; Peck et al., 2012). These results reinforce the hypothesis that warm waters act as a dispersal barrier.

Considering the current antitropical distribution pattern of the genus *Sprattus*, we hypothesize that the lower sea temperatures of the tropics during the cooler glacial periods between the Miocene and Pliocene might have provided a window of opportunity for the most recent common ancestor of *Sprattus* to disperse to the other hemisphere. In this study we test the origin and the monophyly of the genus *Sprattus* using a phylogenetic approach based on DNA sequences from five mitochondrial genome regions (mtDNA). We also examine the historical biogeography of the group, and we used a molecular clock to determine the pattern and timing of species diversification.

## Material and Methods

### Taxon sampling

*Sprattus* species have a least concern status for the IUCN Red List and are not listed under CITES. We did not kill fishes for the purpose of this study; instead, tissue samples were provided by researchers worldwide. Unfortunately, samples for *Sprattus novaehollandiae* were impossible to obtain, therefore we used only three *Sprattus* species from the Southern Hemisphere. All tissue samples arrived fixed in ethanol 90%, and their general capture locations were *S*. *fuegensis* (*n* = 7) from Chilean fjords in the Southeast Pacific Ocean, *S*. *sprattus* (*n* = 5) from Norwegian fjords in the Northeast Atlantic Ocean, *S*. *muelleri* (*n* = 4) from Auckland Harbour, and *S*. *antipodum* (*n* = 1) from Wellington Harbour (New Zealand).

### DNA extraction, PCR and DNA sequencing

Total genomic DNA was dissolved in a buffer containing proteinase K and SDS detergent, and then extracted using a standard phenol-chloroform protocol (Sambrook et al., 1989). DNA was precipitated in 70% ethanol and resuspended in 50 µL of TE buffer. DNA was quantified using a NanoDrop ND-1000 spectrophotometer and diluted to a concentration of 20 ng/µL.

Five mitochondrial fragments were amplified using genus-specific primers (762 bp for Cytochrome b, CytB; 857 bp for Cytochrome Oxidase subunit I, COI; 827 bp for NADH dehydrogenase subunit 2, ND2; and 348 bp for NADH dehydrogenase subunit 3, ND3) designed in this study, and one primer pair described previously (1107 bp for Control Region, CR; Palumbi et al., 1991; Bernatchez, Guyomard & Bonhomme, 1992; see Supporting Information Table S1). The genus-specific primers were designed from the complete mitochondrial genomes sequences deposited in GenBank: *Sprattus sprattus* (NC009593), *S*. *muelleri* (NC016669) and *S*. *antipodum* (NC016673). For CytB, COI, ND2, and ND3 fragments, the PCRs were conducted in 30 µL volumes containing 1X PCR Buffer (Invitrogen^®^; Tris–HCl 200 mM, pH 8,4, KCl, 500 mM), 3 mM MgCl_2_, 0.2 mM of each dNTP’s, 0.2 µM of each primer, 0.4 mg/mL of BSA, 1.5 units of Taq DNA polymerase (Invitrogen^®^), and 2 ng of genomic DNA. Thermal cycling was performed in an MJ Research PTC-200 Thermal Cycler with the following parameters: 95 °C for 180 s, followed by 35 cycles of 94 °C for 30 s, 55 °C for 30 s, 74 °C for 60 s, and a final extension at 74 °C for 300 s. For CR the PCR was amplified using 2 mM MgCl_2_ and the thermo cycling parameters: 94 °C for 300 s, followed by 35 cycles of 94 °C for 30 s, 54 °C for 60 s, 74 °C for 90 s, and a final extension at 74 °C for 600 s. PCR products were purified with ExoSAP-IT^®^ following manufacturer’s guidelines and sequenced in both directions using an ABI 3730xl Genetic Analyzer (Massey University Genome Sequencing Service). Sequences were deposited in GenBank database under the accession numbers MW075156- MW075219. Additional sequences to genus *Sprattus* were included in the ingroup for further analyses: (i) *Clupea harengus* (KC193777) and *Clupea pallasii* (AP009134), including two herring subspecies from *C*. *pallasii* (*C*. *p*. *marisalbi* and *C*. *p*. *suworowi*), given their close relatedness to the genus *Sprattus*; (ii) *Ethmidium maculatum* (AP011602), *Ramnogaster melanostoma* (GQ890211- GQ890214, KU288994 –KU288995), and *Strangomera bentincki* (MW075156-MW075219), given their close relatedness with the *Sprattus*-*Clupea* clade; (iii) *Potamalosa richmondia* (AP011594) and *Hyperlophus vittatus* (AP011593), because they are more distantly related genera to the *Sprattus*-*Clupea* clade; and (iv) *Sprattus sprattus* (AP009234), *Sprattus muelleri* (AP011607), and *Sprattus antipodum* (AP011608) to increase the number of sequences of our target genus. As outgroups, we included *Gilchristella aestuaria* (AP011606) and *Ehirava fluviatilis* (AP011588), two species of the subfamily Ehiravinae used for rooting and time calibration purposes.

Initial alignment was performed in Geneious^®^ 6.0.5 (Kearse et al., 2012), and the final alignment was adjusted by eye. Phylogenetic analyses were conducted separately on each gene (to compare each gene tree) and concatenated fragments (because mitochondrial DNA constitutes a single heritable unit). Divergence time and historical biogeography analyses were conducted using a concatenated alignment of the five mitochondrial fragments. Our concatenated data matrix included 13 sequences (one taxa each) and 3,228 characters.

### Phylogenetic analyses and divergence time

Before conducting the phylogenetic analyses, we performed Xia’s test implemented in DAMBE v5 (Xia et al., 2003; Xia, 2013) to evaluate whether the DNA sequences we used showed evidence of saturation by substitution (*i.e.,* back mutations), which would need to be corrected using a model of sequence evolution during the phylogenetic analyses. We estimated and compared a substitution saturation index with a critical substitution saturation index (Xia et al., 2003; Xia, 2013) to test that the data set is informative for performing phylogenetic analyses. The results of Xia’s test suggest that there is a low level of saturation in our data set, where the critical index of substitution saturation values was significantly higher than the observed index of substitution saturation values (Supporting Information Table S2).

We ran a Bayesian Markov Chain Monte Carlo (BMCMC) phylogenetic analysis that included a general likelihood-based mixture model of gene-sequence evolution and a Reversible-Jump Markov Chain Monte Carlo procedure (Pagel & Meade, 2004; Pagel & Meade, 2006; Pagel & Meade, 2008; Gascuel, 2005). This phylogenetic reconstruction was implemented in BayesPhylogenies v1.1 software (Pagel & Meade, 2004). This approach enables possible models and parameters to be explored, converging towards the model that best fits the data in the sample of posterior trees (Pagel & Meade, 2008). We ran five independent chains using 10^6^ generations, sampling every 10,000th tree sample, and burning the first 25% of the trees. Finally, we obtained the phylogenetic consensus tree using 750 tree samples.

Approximate divergence times among *Sprattus* species were estimated using a Bayesian approach implemented in the BEAST v2 software (Heled & Drummond, 2008; Drummond et al., 2012; Bouckaert et al., 2014). To obtain divergence times, we used the Log-Normal Relaxed Clock Model (LNCM; Drummond et al., 2006; Drummond & Suchard, 2010). We ran this model five times using the most complex sequence evolution model, GTR+I+G, with 10,000,000 generations sampling each 10,000 generations. The outputs of each run were combined in LogCombiner software to increase the Effective Sample Size (ESS) to be at least >200. The ESS of a parameter sampled from an MCMC is the number of effectively independent draws from the posterior distribution of the Markov Chain.

To obtain the posterior distribution of the estimated divergence time, the age of a fossil, †*Lecceclupea ehiravaensis*, dated during the late Campanian in the Late Cretaceous epoch at about 74 MYBP was used. (Taverne, 2011 interpreted this age as part of the Campanian-Maastrichtian; however, 74 MYBP is currently considered within the Campanian according to the ICS International Chronostratigraphic Chart, 2021; http://www.stratigraphy.org.) This age was used as a calibration point to constrain the age in the *Gilchristella aestuaria* and *Ehirava fluviatilis* node. †*Lecceclupea ehiravaensis* has been shown to be a crown member of the clade (*Ehirava*, *Gilchristella*; see Taverne, 2011). Prior age distribution of this clade follows a lognormal distribution using the age boundaries of the geological stage from which the fossil was excavated (*i.e.,* 95% credibility interval). An offset of 74 MYBP was applied to the model. Subsequently, we used the Log-Normal Relaxed Clock Model and previous set parameters to run 10 independent Markov Chain Monte Carlo (MCMC) simulations with a chain length of 10^7^ generations. Sampling was conducted every 10,000 generations and we used as prior distributions the following parameters: the base frequency, proportion invariant sites, and proportions of each transition and transversion, all of those to increase the effective sample size. The individual runs were combined using LogCombiner burning 250 trees per each sample. Finally, a maximum clade credibility tree was created in TreeAnnotator, which enable a summary tree to be visualized in FigTree v1.4 (https://github.com/rambaut/figtree/releases).

### Historical biogeography

We inferred the historical distribution of the genus *Sprattus* and its close relatives using their current distribution (*i.e.,* longitude and latitude as continuous traits). This approach was chosen over the multistate discrete data for the following reasons: (i) discrete data could bias the ancestral state of a descendant species distributed in the same geographical region; (ii) continuous data permit identifying dispersal trends; and (iii) classical discrete multistate estimation does not consider the spherical nature of the earth (O’Donovan, Meade & Venditti, 2018; Gardner, Surya & Organ, 2019; Avaria-Llautureo et al., 2021). For these, we used the current geolocation to infer the ancestral distribution for each node of the phylogenetic tree. To reconstruct the distribution, we used the Geo Model (O’Donovan, Meade & Venditti, 2018) and implemented BayesTraits v3.0 (Pagel & Meade, 2004). The Geo Model estimates the posterior distribution of their geo-position across phylogenetic nodes. We used tree samples obtained in BMCMC phylogenetic analyses and a trait matrix. We ran 10^6^ generations sampled every 10,000 generations to obtain a parameters sample. Posteriorly, a 25% burned-in was used to avoid including parameters sampled before the convergence of the Markov Chain, and a final sample of 750 parameters was obtained. The ancestral distribution of each node was plotted on a paleogeographical perspective using mapast v0.1 R package (Varela & Rothkugel, 2018). We combine paleomaps from 10, 30, 50, 90, 110 MYBP using SETON2012 as a global plate motion model (Seton et al., 2012).

## Results

**Figure 2:**
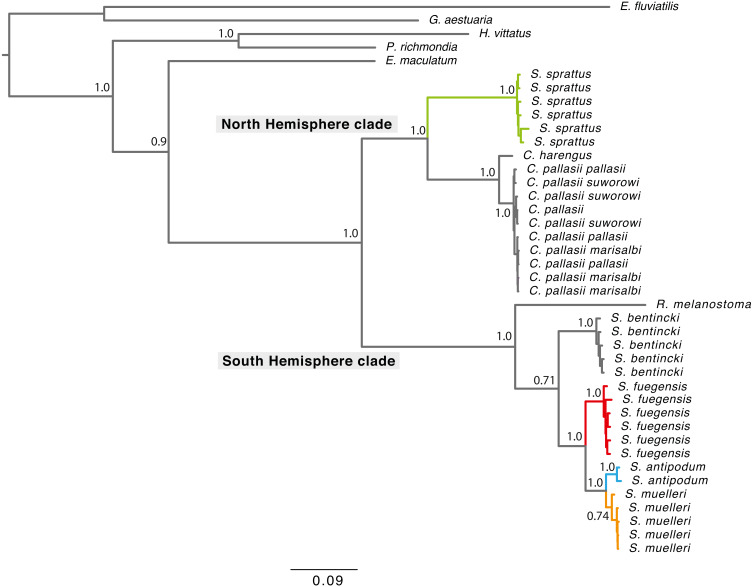
Bayesian consensus tree concatenating of mitochondrial genes from 750 more likely trees. Branch lengths are proportional to the number of substitutions per nucleotide position. Numbers at nodes are posterior probabilities from Bayesian analyses. Grey rectangles indicate current hemisphere distribution. Red branches for *S*. *prattus fuegensis*, orange for S. *muelleri*, light blue for *S*. *antipodum*, and green for *S*. *sprattus*.

Phylogenetic tree reconstructions using the concatenated fragments (Fig. 2) and each mitochondrial fragment independently showed a similar pattern (Fig. S1). Each extant *Sprattus* species forms a monophyletic group. The *Sprattus* species were distributed in the phylogenetic tree in two main clades that matched their antitropical distribution, each in one hemisphere. The Northern Hemisphere clade included *Sprattus sprattus* and the species *Clupea harengus* and *C*. *pallasii* (including their subspecies); the Southern Hemisphere clade included *Sprattus fuegensis*, *S*. *antipodum*, *S*. *muelleri*, *Ramnogaster melanostoma,* and *Strangomera bentincki*. However, overall, the genus *Sprattus* is polyphyletic, because *S*. *sprattus* is closely related to *Clupea* and *S*. *fuegensis*, whereas *S*. *antipodum* and *S*. *muelleri* are closely related to *Ramnogaster* and *Strangomera*.

**Figure 3:**
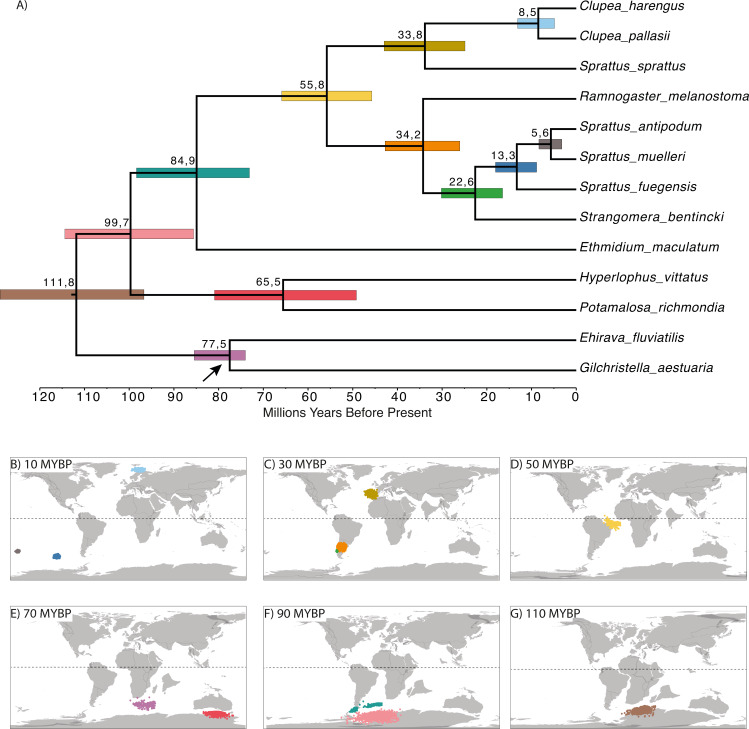
Time-calibrated phylogenetic tree based on Bayesian relaxed-clock analyses (A) and reconstruction of the ancestral geopositioned nodes (B –G). Numbers at nodes are divergence time since the root of each species. Horizontal colored bars indicate the 95% HPD of divergence times, and the scale axis shows divergence times as millions of years ago (MYBP). Analyses based on the topology and branch lengths of the Bayesian phylogenetic trees. Colored dots in B –G correspond to posterior distribution of ancestral locations measured in longitude and latitude. Colors are associated to horizontal-colored bars in (A). Paleomap reconstructions from 10, 30, 50, 90, 110 MYBP were obtained using SETON2012 global plate motion models.

The time-calibrated phylogenetic analyses showed a divergence between Northern and Southern Hemispheres that was dated at 55.8 MYBP (early Eocene; Fig. 3A). There was also another diversification event among the Northern Hemisphere clade at 33.8 MYBP (boundary between Eocene and Oligocene), splitting *Sprattus sprattus* from *Clupea* species. Current species of *Clupea* diverged about 8.5 million years ago (late Miocene). For the Southern Hemisphere clade, species diverged at 33.2 MYBP (early Oligocene). Among the species of the Southern Hemisphere clade, *Strangomera bentincki* split from other *Sprattus* species around 22.6 MYBP (early Miocene), *Sprattus fuegensis* split at 13.3 MYBP (middle Miocene) from their New Zealand relatives, and the most common recent ancestor of *S*. *antipodum* and *S*. *muelleri* diverged around 5.6 MYBP (boundary between Miocene and Pliocene). Ancestral distributions (Figs. 3B–3G) show that the MRCA of the Northern and Southern clades likely inhabited the Southern Hemisphere in the Atlantic Ocean (Fig. 3D).

## Discussion

### Non-monophyletic genus *Sprattus*

We confirmed that the genus *Sprattus* is a polyphyletic group with an antitropical distribution, challenging the taxonomic status of the *Sprattus* species. Considering the two geographic clades in opposing hemispheres, the Northern clade closely relates *S*. *sprattus* with the genus *Clupea*, and the Southern clade closely relates the rest of *Sprattus* members with *Strangomera bentincki* and *Ramnogaster melanostoma*. The relationship among species from the Southern clade has not been described before. This taxonomic incongruence in the genus *Sprattus* has also been identified in studies that use large phylogenies in Clupeiformes and have focused in the identification of the biogeographic or diadromy origin, body size, dispersal pattern, or trophic niche evolution of the group (Lavoué et al., 2013; Bloom & Lovejoy, 2014; Egan et al., 2018; Bloom, Burns & Schriever, 2018; Avaria-Llautureo et al., 2021). Although some of these studies are based on DNA of different types (*i.e.,* mt or nDNA) or taxa (*i.e., Sprattus* members and its close relatives), they support the polyphyly of the genus *Sprattus*. Therefore, our results provide further support for *Sprattus* being polyphyletic and add *S*. *fuegensis* and *Strangomera bentincki* as pieces of the puzzle to understand the evolution in the Southern clade.

Taxonomic classification and phylogenetic relationships among the genera *Sprattus*, *Ramnogaster*, *Strangomera,* and *Clupea* are unclear if they are only based on morphological and meristic traits. All these taxa resemble the *Clupea* type and were first classified as species of *Clupea* ( Whitehead, Smith & Robertson, 1985; Whitehead, 1988). The genus *Sprattus* was erected by Girgensohn (1846) based on *S. haleciformis*, which was later synonymized with *S. sprattus* (Whitehead, 1988), defining the absence of a pterotic bullae as the key diagnostic feature (Mathews, 1884; Whitehead, 1964; Whitehead, 1988; Whitehead, Smith & Robertson, 1985). However, fewer pelvic rays and an anteriorly placed pelvic fin (Whitehead, 1988) have also been used to differentiate *Sprattus* from *Clupea*. The two genera also differentiate in key reproductive traits, whereas *Sprattus* produces pelagic eggs, *Clupea* produces demersal eggs that attach to the seabed or vegetation (Haegele & Schweigert, 1985; Whitehead, 1988). Finally, the genera *Sprattus* and *Ramnogaster* share the absence of pterotic bullae, but differ in fin-ray numbers, whereas *Sprattus* differs from *Strangomera* on having more gill rakers (Whitehead, 1988).

Incomplete sorting lineage, introgression, or convergence of morphological traits could be plausible explanations for the current *Sprattus* taxonomic classification and our gene tree. The first two can be ruled out, because none of the species of this study shared or had similar haplotypes. Introgression may also be ruled out, because the fishes have different reproductive strategies: pelagic or demersal eggs (Haegele & Schweigert, 1985; Whitehead, 1988), so there is little opportunity for cross-fertilization. However, introgression could be true if divergence in reproductive ecology occurred at an initial stage older than 33.8 MYBP between *S*. *sprattus* and *Clupea* species. Introgression and ancient hybridization events could be identified by comparing mtDNA and nDNA (Saitoh et al., 2011), however, this has not been detected in clupeid phylogenies (Bloom & Lovejoy, 2014). We cannot discard the convergence of morphological traits explanation given that there are traits that look similar and others that support the separation of *Sprattus* and *Clupea* (Mathews, 1884; Whitehead, 1964; Whitehead, 1988; Whitehead, Smith & Robertson, 1985).

For *Sprattus* species from the Southern Hemisphere, we found that *S*. *fuegensis* from South America is the sister to New Zealand’s sympatric *S*. *antipodum* and *S*. *muelleri*. Nonetheless, we need to keep in mind that we could not include *S*. *novaehollandiae*, hence further studies should include this species. For New Zealand sprats, it only has been suggested that these species might have different ecological requirements considering their sympatry (Whitehead, Smith & Robertson, 1985). We suggest that further investigations be done to disentangle the mechanisms that promoted sympatric speciation for *S*. *antipodum* and *S*. *muelleri*.

### Divergence time and historical biogeography

The results based on a fossil calibration showed that the two antitropical clades diverged in the Eocene (55.8 MYBP; older than we hypothesized), with a likely origin in the Atlantic Ocean in the Southern Hemisphere. The species within the Northern Hemisphere clade diverged at 33.8 and in the Southern Hemisphere at 33.4 MYBP, during the early Oligocene. Cheng & Lu (2006) and Jérôme et al. (2003) estimated that the divergence event of the two genera occurred between 6.75–2.66 MYBP (late Neogene-early Quaternary). This estimation disagrees with the older divergence time found in our study, which could be explained by the calibrating method used by the authors. Different calibrating methods typically yield different results, and each method has its own particular challenges. In previous studies the authors used a standard nucleotide substitution rate for fish, which is a method that depends on the timescale over which those rates are measured (Hipsley & Müller, 2014) and could generate an overestimation of divergence times (Phillips, 2009; Ho et al., 2011; Hipsley & Müller, 2014). Fossil calibrations do not produce this problem, although the uncertainty in age and phylogenetic position present a different challenge (Hipsley & Müller, 2014). To address this and avoid the overestimation of the divergence time, we ran our analysis based on the fossilized birth-death process calibration method and a Bayesian framework, which included the uncertainty of dating species divergences and yield with more accurate node age estimates (Heath, Huelsenbeck & Stadler, 2014; Bouckaert et al., 2014; Gavryushkina et al., 2017).

The Eocene was the warmest geological epoch of the last 65 million years (Zachos et al., 2001), where sea surface temperatures in the Atlantic tropical areas may have been up to 38 °C (Cramwinckel et al., 2018). The ancestor of Clupeoidei originated and diversified in the tropical Indo-West Pacific region during the Lower Cretaceous (119 MYBP, Lavoué et al., 2013), and it would have been adapted to warm, marine temperatures (i.e., >25 °C; Lavoué et al., 2013; Bloom & Lovejoy, 2014). Considering this, our analyses show that the Clupeidae lineage spread to the Southern Hemisphere earlier than the clades that included *Sprattus*, *Clupea* and close relatives. Similarly, the species of *Potamalosa*, *Hyperlophus* and *Ethmidium* also inhabit temperate waters in the Southern Hemisphere, suggesting that this old south-distributed group of fishes was able to cross the tropics but not to adapt to the warmer environment. Nonetheless, extant members of the genera *Sprattus* and *Clupea* are now distributed antitropically in much colder temperate waters (Whitehead, Smith & Robertson, 1985; Lavoué et al., 2013), and although they mainly inhabit marine environments (Bloom & Lovejoy, 2014), they can also inhabit areas with highly variable environments, such as fjords (e.g., Glover et al., 2011; Canales-Aguirre et al., 2016; Canales-Aguirre et al., 2018).

Antitropical distribution patterns are traditionally explained by dispersal and vicariance mechanisms (Stepien & Rosenblatt, 1996; Grant & Bowen, 1998; Burridge, 2002; Le Port, Pawley & Lavery, 2013). Dispersalists have proposed several hypotheses to explain dispersal across the tropics: island integration (Rotondo et al., 1981), dispersal at shallow depths during glaciations (Lindberg, 1991), and isothermal submergence (Hubbs, 1952). Island integration refers to the formation of endemic biotas through the movement of individuals using islands or seamounts (Rotondo et al., 1981). In our case, we can discard this explanation, because clupeids are typically marine and inhabit productive coastal areas (Whitehead, 1988). Dispersal at shallow depths during glaciations is a well-recognized dispersal mechanism for several pelagic fishes during the Pleistocene (Burridge & White, 2000; Burridge, 2002; Grant, Leslie & Bowen, 2005). The isothermal submergence hypothesis refers to the possibility that marine organisms adapted to cool or temperate areas are able to disperse across the tropical region through deeper, colder tropical waters (Hubbs, 1952). Taking into account that the MRCA of these two clades diversified in the warm Eocene, and then each clade diversified between the late Eocene and early Oligocene epochs the isothermal submergence hypothesis seems to be the most plausible explanation. This later because the temperatures begin to decrease until initiation of Antarctic glaciation (Zachos et al., 2001) and some clupeoids, such as herrings, may dive as much as 200 m (Blaxter, Denton & Gray, 1981). Vicariant mechanisms such as plate tectonic, relictual distribution, and equatorial isolation by climatic change or biological interactions have been advocated by others studies (Stepien & Rosenblatt, 1996; Saitoh et al., 2011). However, mechanisms associated with plate tectonics are not supported by our results, because the divergence time among nominal species of *Sprattus* and *Clupea* would have occurred during the Eocene, and the present continental configuration closely resembles the configuration of the continents during that time. Studies, such as those by Grant & Bowen (1998) and Grant, Leslie & Bowen (2005) on marine pelagic fishes have supported a dispersalist mechanism to explain the antitropical distribution and exclude vicariant explanations as well.

Dispersion from their ancestral habitat involved adaptation to colder waters, while simultaneously expanding their tolerance to fluctuations in salinity, allowing them to also colonize low saline habitats. The warmer equatorial waters have remained as a key barrier to dispersal between hemispheres, which has only been crossed when windows of colder environments appeared across the tropics or, more plausibly, by using deeper, colder tropical waters as proposed by the isothermal submergence hypothesis.

##  Supplemental Information

10.7717/peerj.11737/supp-1 Supplemental Information 1 Primers used in this study

10.7717/peerj.11737/supp-2 Supplemental Information 2 Comparison of the index of substitution saturation (ISS) with the critical index of substitution saturation (ISSc) that defines a threshold for significant saturation in the data for symmetrical and asymmetrical tree topology

10.7717/peerj.11737/supp-3 Supplemental Information 3 Bayesian consensus tree of each mitochondrial genes and concatenated data Branch lengths are proportional to the number of substitutions per nucleotide position. Numbers at nodes are posterior probabilities from Bayesian analyses.

10.7717/peerj.11737/supp-4 Supplemental Information 4 Sequences used in this study

## Additional Information and Declarations

### Data Availability

The following information was supplied regarding data availability: The sequences are available at NCBI: MW075156–MW075219, and MW075156–MW075219, and the raw measurements are available in the Supplementary File.

## References

[ref-1] Aranis A, Meléndez R, Pequeño G, Cerna F (2007). *Sprattus fuegensis* en aguas interiores de Chiloé, Chile (Osteichthyes: Clupeiformes: Clupeidae). Gayana.

[ref-2] Avaria-Llautureo J, Venditti C, Rivadeneira MM, Inostroza-Michael O, Rivera RJ, Hernández CE, Canales-Aguirre CB (2021). Historical warming consistently decreased size, dispersal and speciation rate of fish. Nature Climate Change.

[ref-3] Bernatchez L, Guyomard R, Bonhomme F (1992). DNA sequence variation of the mitochondrial control region among geographically and morphologically remote European brown trout *Salmo trutta* populations. Molecular Ecology.

[ref-4] Blaxter JHS, Denton EJ, Gray JAB, Tavolga WN, Popper AN, Fay RR (1981). Acousticolateralis system in clupeid fishes. Hearing and sound communication in fishes.

[ref-5] Bloom DD, Burns MD, Schriever TA (2018). Evolution of body size and trophic position in migratory fishes: a phylogenetic comparative analysis of Clupeiformes (anchovies, herring, shad and allies). Biological Journal of the Linnean Society.

[ref-6] Bloom DD, Lovejoy NR (2014). The evolutionary origins of diadromy inferred from a time-calibrated phylogeny for Clupeiformes (herring and allies). Proceedings of the Royal Society B: Biological Sciences.

[ref-7] Bouckaert R, Heled J, Kühnert D, Vaughan T, Wu C-H, Xie D, Suchard MA, Rambaut A, Drummond AJ (2014). BEAST 2: a software platform for Bayesian evolutionary analysis. PLOS Computational Biology.

[ref-8] Bowen BW, Grant WS (1997). Phylogeography of the sardines (*Sardinops* spp.): assessing biogeographic models and population histories in temperate upwelling zones. Evolution.

[ref-9] Burridge CP (2002). Antitropicality of Pacific fishes: molecular insights. Environmental Biology of Fishes.

[ref-10] Burridge CP, White RWG (2000). Molecular phylogeny of the antitropical subgenus *Goniistius* (Perciformes: Cheilodactylidae: *Cheilodactylus*): evidence for multiple transequatorial divergences and non-monophyly. Biological Journal of the Linnean Society.

[ref-11] Canales-Aguirre CB, Ferrada-Fuentes S, Galleguillos R, Hernández CE (2016). Genetic structure in a small pelagic fish coincides with a marine protected area: seascape genetics in Patagonian fjords. PLOS ONE.

[ref-12] Canales-Aguirre CB, Ferrada-Fuentes S, Galleguillos R, Oyarzún FX, Buratti CC, Hernández CE (2018). High genetic diversity and low-population differentiation in the Patagonian sprat (*Sprattus fuegensis*) based on mitochondrial DNA. Mitochondrial DNA Part A.

[ref-13] Cheng QQ, Lu DR (2006). Phylogenetic analysis and relative-rate test of nine Clupeidae fishes Osteichthyes Clupeiformes inferred from cytochrome b gene sequence of mitochondrial DNA. Marine Fisheries.

[ref-14] Cramwinckel MJ, Huber M, Kocken IJ, Agnini C, Bijl PK, Bohaty SM, Frieling J, Goldner A, Hilgen FJ, Kip EL, Peterse F, van der Ploeg R, Röhl U, Schouten S, Sluijs A (2018). Synchronous tropical and polar temperature evolution in the Eocene. Nature.

[ref-15] Drummond AJ, Ho SYW, Phillips MJ, Rambaut A (2006). Relaxed phylogenetics and dating with confidence. PLOS Biology.

[ref-16] Drummond AJ, Suchard MA (2010). Bayesian random local clocks, or one rate to rule them all. BMC Biology.

[ref-17] Drummond AJ, Suchard MA, Xie D, Rambaut A (2012). Bayesian phylogenetics with BEAUti and the BEAST 1.7. Molecular Biology and Evolution.

[ref-18] Egan JP, Bloom DD, Kuo C-H, Hammer MP, Tongnunui P, Iglésias SP, Sheaves M, Grudpan C, Simons AM (2018). Phylogenetic analysis of trophic niche evolution reveals a latitudinal herbivory gradient in Clupeoidei (herrings, anchovies, and allies). Molecular Phylogenetics and Evolution.

[ref-19] Frederiksen M, Edwards M, Richardson AJ, Halliday NC, Wanless S (2006). From plankton to top predators: bottom-up control of a marine food web across four trophic levels. Journal of Animal Ecology.

[ref-20] Fricke R, Eschmeyer W, Van der Laan R (2021). Eschmeyer’s catalog of fishes: genera, species, references. http://researcharchive.calacademy.org/research/ichthyology/catalog/fishcatmain.asp.

[ref-21] Gardner JD, Surya K, Organ CL (2019). Early tetrapodomorph biogeography: controlling for fossil record bias in macroevolutionary analyses. Comptes Rendus Palevol.

[ref-22] Gascuel O (2005). Mathematics of evolution and phylogeny.

[ref-23] Gavryushkina A, Heath TA, Ksepka DT, Stadler T, Welch D, Drummond AJ (2017). Bayesian total-evidence dating reveals the recent crown radiation of penguins. Systematic Biology.

[ref-24] Glover KA, Skaala Ø, Limborg M, Kvamme C, Torstensen E (2011). Microsatellite DNA reveals population genetic differentiation among sprat (*Sprattus sprattus*) sampled throughout the Northeast Atlantic, including Norwegian fjords. ICES Journal of Marine Science.

[ref-25] Grant WS (1986). Biochemical genetic divergence between Atlantic, *Clupea harengus*, and Pacific, *C. pallasi*, herring. Copeia.

[ref-26] Grant WS, Bowen BW (1998). Shallow population histories in deep evolutionary lineages of marine fishes: insights from sardines and anchovies and lessons for conservation. Journal of Heredity.

[ref-27] Grant WS, Lecomte F, Bowen BW (2010). Biogeographical contingency and the evolution of tropical anchovies (genus *Cetengraulis*) from temperate anchovies (genus *Engraulis*). Journal of Biogeography.

[ref-28] Grant WS, Leslie RW (1996). Late Pleistocene dispersal of Indian-Pacific sardine populations in an ancient lineage of the genus *Sardinops*. Marine Biology.

[ref-29] Grant WS, Leslie RW, Bowen BW (2005). Molecular genetic assessment of bipolarity in the anchovy genus *Engraulis*. Journal of Fish Biology.

[ref-30] Haegele CW, Schweigert JF (1985). Distribution and characteristics of herring spawning grounds and description of spawning behavior. Canadian Journal of Fisheries and Aquatic Sciences.

[ref-31] Heath TA, Huelsenbeck JP, Stadler T (2014). The fossilized birth-death process for coherent calibration of divergence-time estimates. Proceedings of the National Academy of Sciences of the United States of America.

[ref-32] Heled J, Drummond AJ (2008). Bayesian inference of population size history from multiple loci. BMC Evolutionary Biology.

[ref-33] Hipsley CA, Müller J (2014). Beyond fossil calibrations: realities of molecular clock practices in evolutionary biology. Frontiers in Genetics.

[ref-34] Ho SYW, Lanfear R, Bromham L, Phillips MJ, Soubrier J, Rodrigo AG, Cooper A (2011). Time-dependent rates of molecular evolution. Molecular Ecology.

[ref-35] Hubbs CL (1952). Antitropical distribution of fishes and other organisms. Proceedings of the 7th Pacific Science Congress.

[ref-36] Jérôme M, Lemaire C, Bautista JM, Fleurence J, Etienne M (2003). Molecular phylogeny and species identification of sardines. Journal of Agricultural and Food Chemistry.

[ref-37] Kearse M, Moir R, Wilson A, Stones-Havas S, Cheung M, Sturrock S, Buxton S, Cooper A, Markowitz S, Duran C, Thierer T, Ashton B, Meintjes P, Drummond A (2012). Geneious Basic: an integrated and extendable desktop software platform for the organization and analysis of sequence data. Bioinformatics.

[ref-38] Lavoué S, Miya M, Musikasinthorn P, Chen W-J, Nishida M (2013). Mitogenomic evidence for an Indo-West Pacific origin of the Clupeoidei (Teleostei: Clupeiformes). PLOS ONE.

[ref-39] Lavoué S, Miya M, Saitoh K, Ishiguro NB, Nishida M (2007). Phylogenetic relationships among anchovies, sardines, herrings and their relatives (Clupeiformes), inferred from whole mitogenome sequences. Molecular Phylogenetics and Evolution.

[ref-40] Le Port A, Pawley MDM, Lavery SD (2013). Speciation of two stingrays with antitropical distributions: low levels of divergence in mitochondrial DNA and morphological characters suggest recent evolution. Aquatic Biology.

[ref-41] Li C, Ortí G (2007). Molecular phylogeny of Clupeiformes (Actinopterygii) inferred from nuclear and mitochondrial DNA sequences. Molecular Phylogenetics and Evolution.

[ref-42] Lindberg DR (1991). Marine biotic interchange between the northern and southern hemispheres. Paleobiology.

[ref-43] Martínez-Porchas M (2009). Thermal behavior of the Pacific sardine (*Sardinops sagax*) acclimated to different thermal cycles. Journal of Thermal Biology.

[ref-44] Mathews D (1884). Report on sprat fishing during the winter of 1883-84. Report of the Fishery Board for Scotland.

[ref-45] O’Donovan C, Meade A, Venditti C (2018). Dinosaurs reveal the geographical signature of an evolutionary radiation. Nature Ecology & Evolution.

[ref-46] Pagel M, Meade A (2004). A phylogenetic mixture model for detecting pattern-heterogeneity in gene sequence or character-state data. Systematic Biology.

[ref-47] Pagel M, Meade A (2006). Bayesian analysis of correlated evolution of discrete characters by reversible-jump Markov chain Monte Carlo. American Naturalist.

[ref-48] Pagel M, Meade A (2008). Modelling heterotachy in phylogenetic inference by reversible-jump Markov chain Monte Carlo. Philosophical Transactions of the Royal Society B: Biological Sciences.

[ref-49] Palumbi SR, Martin A, Romano S, McMillan WO, Stice L, Grabowski G (1991). Simple fool’s guide to PCR.

[ref-50] Peck MA, Kanstinger P, Holste L, Martin M (2012). Thermal windows supporting survival of the earliest life stages of Baltic herring (*Clupea harengus*). ICES Journal of Marine Science.

[ref-51] Phillips MJ (2009). Branch-length estimation bias misleads molecular dating for a vertebrate mitochondrial phylogeny. Gene.

[ref-52] Rotondo GM, Springer VG, Scott GAJ, Schlanger SO (1981). Plate movement and island integration—a possible mechanism in the formation of endemic biotas, with special reference to the Hawaiian islands. Systematic Biology.

[ref-53] Saitoh K, Sado T, Doosey MH, Bart Jr HL, Inoue JG, Nishida M, Mayden RL, Miya M (2011). Evidence from mitochondrial genomics supports the lower Mesozoic of South Asia as the time and place of basal divergence of cypriniform fishes (Actinopterygii: Ostariophysi). Zoological Journal of the Linnean Society.

[ref-54] Sambrook J, Fritsch EF, Maniatis T (1989). Molecular cloning: a laboratory manual.

[ref-55] Seton M, Müller RD, Zahirovic S, Gaina C, Torsvik T, Shephard G, Talsma A, Gurnis M, Turner M, Maus S, Chandler M (2012). Global continental and ocean basin reconstructions since 200 Ma. Earth-Science Reviews.

[ref-56] Stepien CA, Rosenblatt RH (1996). Genetic divergence in antitropical pelagic marine fishes (*Trachurus*, *Merluccius*, and *Scomber*) between North and South America. Copeia.

[ref-57] Taverne L (2011). Les Poissons crétacés De Nardò, 33°. *Lecceclupea ehiravaensis* gen. et sp. nov. (Teleostei, Clupeidae). Bollettino Del Museo Civico di Storia Naturale di Verona.

[ref-58] Varela S, Rothkugel S (2018). https://github.com/macroecology/mapast.

[ref-59] Whitehead PJP (1964). A new genus and subgenus of clupeid fishes and notes on the genera *Clupea*, *Sprattus* and *Clupeonella*. Annals and Magazine of Natural History.

[ref-60] Whitehead PJP (1988). FAO species catalogue: an annotated and illustrated catalogue of the Herrings, Sardines, Pilchards, Sprats, Shads, Anchovies and Wolf-Herrings.

[ref-61] Whitehead PJP, Smith PJ, Robertson DA (1985). The two species of sprat in New Zealand waters (*Sprattus antipodum* and *S. muelleri*). New Zealand Journal of Marine and Freshwater Research.

[ref-62] Wilson AB, Teugels GG, Meyer A (2008). Marine incursion: the freshwater herring of Lake Tanganyika are the product of a marine invasion into west Africa. PLOS ONE.

[ref-63] Xia X (2013). DAMBE5: a comprehensive software package for data analysis in molecular biology and evolution. Molecular Biology and Evolution.

[ref-64] Xia X, Xie Z, Salemi M, Chen L, Wang Y (2003). An index of substitution saturation and its application. Molecular Phylogenetics and Evolution.

[ref-65] Zachos J, Pagani M, Sloan L, Thomas E, Billups K (2001). Trends, rhythms, and aberrations in global climate 65 Ma to present. Science.

