# Supplementary material for: Phylogenetic relationships, origin and historical biogeography of the genus Sprattus (Clupeiformes: Clupeidae)
Source: PeerJ. 2021 Aug 18;9:e11737. doi: 10.7717/peerj.11737 (PMC8380030; doi:10.7717/peerj.11737)
Supplement: Supplemental Information 1 [file peerj-09-11737-s001.docx]

Supporting Information Table S1: Primers used in this study.

| **Gene** | **Acronym** | **Primer name** | **5'-3'** | **Reference** |
| --- | --- | --- | --- | --- |
| Cytochrome b | CytB | SprCb1F | TTTCCACCCGGACTCTAACC | This study |
|  |  | SprCb1R | CTCCGGATTACAAAACCGGC |  |
|  |  |  |  |  |
|  |  | SprCb2F | GACTCGAAAAACCACCGTTGT | This study |
|  |  | SprCb2R | TGGGAGTTAGGGGTGGAAGT |  |
|  |  |  |  |  |
| NADH dehydrogenase | Nd2 | SprNd2F | CATACCCCGGACATGTTGGT | This study |
|  |  | SprNd2R | CTGCTTGGAGCTTTGAAGGC |  |
|  |  |  |  |  |
| NADH dehydrogenase 3 | Nd3 | SprNd3F | GACAGTACAGGTGGCTTCCA | This study |
|  |  | SprNd3R | GGGCCGAAACCAGAAGTCTT |  |
|  |  |  |  |  |
| Cytochrome Oxidase subunit I | COI | SprCOIF | GCTACAATCCGCCGCCTAA | This study |
|  |  | SprCOIR | GGGGTTCGATTCCTCCCTTT |  |
|  |  |  |  |  |
| Control Region (D-loop) | CR | L19 (modified) | CCACTAGCTCCCAAAGC | Bernatchez et al., 1992 |
|  |  | 12Sar | ATAGTGGGGTATCTAATCCCAGTT | Palumbi et al., 1991 |
