# Supplementary material for: Phylogenetic relationships, origin and historical biogeography of the genus Sprattus (Clupeiformes: Clupeidae)
Source: PeerJ. 2021 Aug 18;9:e11737. doi: 10.7717/peerj.11737 (PMC8380030; doi:10.7717/peerj.11737)
Supplement: Supplemental Information 2 [file peerj-09-11737-s002.docx]

Supporting Information Table S2: Comparison of the index of substitution saturation (ISS) with the critical index of substitution saturation (ISSc) that defines a threshold for significant saturation in the data for symmetrical and asymmetrical tree topology.

| **Gene** | **Iss** | **Iss.c (Symmetrical)** | **Iss.c (Asymmetrical)** | **d.f.** | **Probability** |
| --- | --- | --- | --- | --- | --- |
| CytB | 0,1512 | 0,752 | 0,4909 | 761 | <0.00001 |
| Nd2 | 0,1688 | 0,7564 | 0,4926 | 826 | <0.00001 |
| Nd3 | 0,1492 | 0,6904 | 0,4823 | 347 | <0.00001 |
| COI | 0,103 | 0,7551 | 0,5457 | 856 | <0.00001 |
| CR | 0,3759 | 0,7722 | 0,5347 | 1106 | <0.00001 |
| Concatenated | 0,2035 | 0,8096 | 0,627 | 3900 | <0.00001 |
